# The effect of two sessions of combined jump and sprint training per week on fitness parameters in soccer players. A randomized controlled trial

**DOI:** 10.5114/biolsport.2023.119287

**Published:** 2022-09-22

**Authors:** Mattia Bianchi, Liam Anderson, Thomas E Brownlee, Lorenzo Bossi, Marco Beato

**Affiliations:** 1 School of Health and Sports Science, University of Suffolk, Ipswich, United Kingdom; 2 School of Sport, Exercise and Rehabilitation Sciences, University of Birmingham, Birmingham, United Kingdom

**Keywords:** Football, Team sports, Performance, Training, Jumps

## Abstract

This study aimed to investigate the effect of a combined jump and sprint training program, two sessions a week for 6 weeks, on sprinting, change of directions (COD) and jumping performance in semi-professional soccer players. Twenty soccer players were enrolled in this randomized controlled trial (age 20 ± 2 years, body mass 74.3 ± 5.9 kg). Players were randomized into two groups such as training group (TG, n = 10 players) or control group (CG, n = 10 players). Physical tests were performed before and after 6 weeks of training such as sprint 10 m, sprint 30 m, 505-COD test and standing long jump (LJ). The two groups performed the same training except for the combined jump and sprint training performed twice a week by TG. After 6 weeks of training, between-group analysis reported statistical difference in favor of the TG in sprint 10 m (p = 0.015, η^2^ = 0.295, *large*), sprint 30 m (p < 0.001, η^2^ = 0.599, *large*), in 505-COD (p = 0.026, η^2^ = 0.154, *large*), and LJ (p = 0.025, η^2^ = 0.027, *small*). These data indicate that combined sprint and jump training, when performed twice a week, for the duration of 6 weeks, in addition to the regular team training, can improve specific physical performance in male soccer players. This study has shown that a volume increment of 10% after 3 weeks of training can be a suitable training dose progression and that a combination of 64–70 jumps and 675–738 m of sprinting training per session can yield benefits in sprint, COD and jump performance.

## INTRODUCTION

Soccer is an intermittent sport where brief bouts of high-intensity linear and multi-directional activity are superimposed on a larger background of low-intensity exercise [1–3]. The physical demands of soccer match play have been well investigated, with players typically covering distances of 10–13 km and performing approximately 1,350 high-intensity actions, such as sprints, accelerations, decelerations, and changes of direction (COD) [1]. Such high-intensity movements are an important ability to acquire to improve performance as they are related to playing standard and high-reward situations in soccer match play (e.g., scoring a goal, defending a goal) [4, 5]. Moreover, high-intensity demands have evolved over time with distances and actions increasing from 2006–2013 in the English Premier League [6], which highlights the importance of football-specific training [2, 7].

The development of short-distance sprint performance and COD are a vital component of athletic performance within the football codes [8–10]. Sprint training exposes the body to large forces, which may elicit coordinative and neuromuscular adaptations [11–13]. Although regular sprint training exposure is expected to facilitate adaptations to improve sprinting performance in athletes, sprint training alone seems not so effective in developing short sprint performance in football code athletes [8]. This could be due to the high sprint demands of the game eliciting players’ high chronic adaptations. Therefore, soccer practitioners should research the most suitable training method combinations to perform alongside sprint training in order to achieve their aims [14–17]. For instance, previous research reported that combining both sprint and jumping training in youth soccer players elicits positive fitness adaptations [18] as well as the combination of jump and COD training [14]. This is further supported from a recent meta-analysis that has identified that primary methods alone (e.g., sprint technique, sprinting) are ineffective in eliciting adaptations (i.e., sprint < 20 m) [8], while combining primary methods with either secondary (e.g., resisted, or assisted sprinting) or tertiary (e.g., strength training and plyometrics) can induce positive adaptative responses.

Jump training is a popular and effective method to improve power and sprint performance [19]. It involves jumping exercises that elicits the stretch-shortening cycle (SSC) muscle action and enhances the ability of the neural and musculoskeletal system to produce maximal force in the shortest possible time [19, 20]. SSC based movements are a natural part of most sporting movements and are regularly performed within soccer training and match play [19, 21]. A previous systematic review has showed clear benefits of implementing jump (plyometric) training into the increasing muscle power in 13 out of 16 studies analyzed, with positive effects ranging between 2.4 and 31.3% [19]. Additionally, research found that jump training when performed once and twice a week can increase sport specific physical capacities in soccer players such as horizontal and vertical jump and short distance sprint performance [22]. Therefore, the implementation of jump training, as a standalone method or combined within a training program, is a valid training method to elicit sport specific physical adaptations in soccer players [23], however, to maximize soccer specific capacities, utilizing a combination of jumping, sprinting and COD exercises in an integrated program seems worthwhile [14, 19, 24, 25].

Previous research combining sprinting and other training methods into a soccer training program produced meaningful fitness adaptations [8], which cannot be achieved with only sprint training or regular soccer training, however, the current evidence available in the scientific literature is limited, in particular when training programs are implemented in season [24], and with a robust research design (i.e., randomized controlled trial) [21]. Therefore, this study aimed to investigate the effect of a combined jump and sprint training program, two session a week for 6 weeks (within regular team training), on players sprinting, COD and jumping performance in semi-professional soccer players. We hypothesize that the addition of this training program would improve the players’ sport specific performance indicators compared to the control group, which performed the usual soccer training program.

## MATERIALS AND METHODS

### Participants

Twenty semi-professional male soccer players were enrolled in this study (mean ± SD; age 20 ± 2 (range 18–22) years, body mass 74.3 ± 5.9 kg, height 1.77 ± 6.1 m). Only outfield players were included in this study, while goalkeepers and under 18 players were excluded as well injured players. The Ethics Committee of the University of Suffolk (UK) approved this study (RETH19/020). All procedures were conducted according to the Declaration of Helsinki for human studies. All subjects were informed about the potential risks and benefits of the study and signed a written informed consent.

### Experimental design

**FIG. 1:**
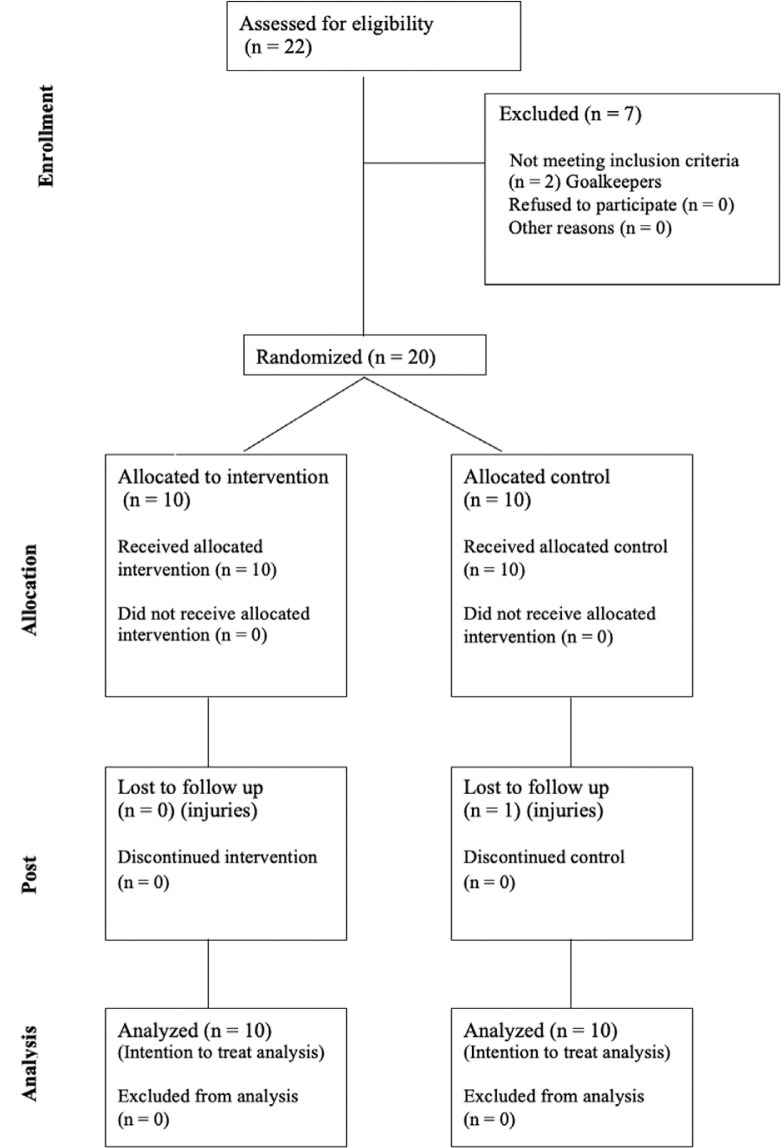
CONSORT diagram showing the flow of participants through each stage of a randomized controlled trial.

Using a randomized controlled design, this study examined the effect of 6 weeks of combined jump and sprint training (twice a week) on sprint, COD and jump performance in a sample of semi-professional male soccer players. Authors calculated *a priori* the statistical power of this study using G*Power (version 3.1.9.7, Heinrich Heine University Düsseldorf, Germany). A sample size of 20 participants was necessary to have a sufficient power (> 0.80) based on alpha selected (5%) and *moderate* effect size (f = 0.35). For such a reason, 20 players were enrolled in this study with an actual power of 0.841. Before the beginning of the protocol, researchers performed a randomization according to a computer-generated sequence (https://www.sealedenvelope.com/simple-randomiser/v1/lists). As reported in the CONSORT flow (Figure 1), after the randomization, players were allocated to either a training group (TG, n = 10 players) or control group (CG, n = 10 players).

Nineteen players finished the protocol, while one player of the CG did not because of an injury that occurred during a match. An intention to treat analysis was used in this study, therefore researchers included in the statistical analysis all the players (n = 20) initially enrolled in this study therefore the final actual power of the study was > 0.8 [26, 27].

### Experimental procedure

The players enrolled in this study had prior experience of the test battery being used as it was part of their normal evaluation routine with the club, so specific testing familiarization was not required. A standard warm-up routine was performed before each testing session (duration 10 min), which included low-intensity running, dynamic stretching (static stretching was avoided) and sport-specific movements that allowed for the preparation of the players to perform jump and spring sessions. Players performed sprint tests of 10 m and 30 m distance, which were used to evaluate the effect of the combined jump and sprint training on linear sprint ability [14]. Infrared timing gates (Microgate, Bolzano, Italy) were placed at the start and end of each of the mentioned distances. The players performed each test three times with 2 min of recovery between the sprints and the best result in each assessment was considered for the data final analysis. The reliability of these tests was previously reported [24] and confirmed in this study; intraclass coefficient correlation (ICC) = 0.88 (*good*) and 0.92 (*excellent*), respectively.

The variation of COD ability after the training protocol was assessed by a 505-COD test. The reliability of this protocol was previously reported [28] and confirmed in this study (ICC = 0.91, *excellent*). On the starting command, the players sprinted for the distance of 15 m. Timing gates were positioned at 10 m and the players passed through it. Players turned on their dominant limb and sprinted back through the timing gates.

A standing long jump test (LJ) was used to evaluate the players’ power capacity of the leg muscles following the 6 weeks of intervention. LJ was used to evaluate improvement of horizontal non-rebounding ability, which has been previously described as a reliable assessment [14, 21] – ICC in this study was 0.95, *excellent*. Players performed three trials of a maximal bilateral jump with free arms. The jump distance was measured using a measuring tape from the starting line to the point at which the heel contacted the ground on landing.

Researchers asked the players to avoid intake of alcohol and caffeine 24 hours before the tests, as well as to maintain their normal nutritional routine during the protocol period. To avoid any circadian effect on physical data, both assessments and training protocol were performed at the same time of the day (from 15.00 to 17.00).

### Training program

The combined jump and sprint training utilized in the current protocol is supported by previous evidence [8, 22]. Previous research reported that short-term training protocols are generally not appropriate to obtain significant variations [15], while longer duration protocols (e.g., 6 weeks or longer) are generally suitable to obtain some significant and meaningful fitness variations in soccer players [8, 29]. Moreover, two sessions a week are generally needed to obtain such variation in season, while previous studies reported that a single session a week is generally a training frequency too low to offer such variations [24]. The team enrolled in this study performed 3 sessions a week, and one match; players of the two groups performed the same training except for the combined jump and sprint training performed by the TG.

Training was designed with the sport scientists of the soccer club involved in this study, which considered the period of the season and the aims of the club. Two training session a week were considered adequate for the period of the season (in-season) in line with previous literature [8, 14]. Training intervention was divided into two parts, where the first three weeks utilized a specific jumps and sprinting volume, which was increased from the fourth week of 10%. TG undertook the program reported in table 1. Tapering procedure was not planned in the last week of the protocol, however at least 72 h of recovery separated the last training session from the testing session. A standard warm-up routine was performed before each training session (10 to 12 min), which included low-intensity running, dynamic stretching (static stretching was avoided) and sport-specific movements that allowed for the preparation of the players to perform jump and spring sessions. The intensity required during jumps and sprints was maximal – S&C coaches of the team incited the players during the conditioning sessions. Each group (TG and CG) was always coached by a minimum of one qualified S&C coach during the protocol (minimum players/coach ratio was 10/1).

**TABLE 1:** Training programme.

Weeks	Training Programme
1 – 3 weeks	- 10 cm hurdle jumps (4 jumps) + 5 m sprint (× 5 sets) - Split squat jump + 10 cm hurdle jumps (5 jumps) + 5 m sprint (× 4 sets) - Linear sprint 30 m (3 repetitions) (× 7 sets)
	The recovery between sets was 20–30 seconds, recovery between exercises was 2 minutes
	Total jumps: 64 jumps per session Total sprint distance: 675 m per session

4 – 6 weeks	- 10 cm hurdle jumps (5 jumps) + 5 m sprint (× 5 sets) - Split squat jump + 10 cm hurdle jumps (6 jumps) + 5 m sprint (× 4 sets) - Linear sprint 33 m (3 repetitions) (× 7 sets)
	The recovery between sets was 20–30 seconds, recovery between exercises was 2 minutes
	Total jumps: 70 jumps per session Total sprint distance: 738 m per session
	(10% increment compared to the first training block)

The CG did a recovery session composed of low intensity technical with the coach. CG did not perform any additional jump or sprint training to compensate for what was performed by the TG. Both TG and CG did the same soccer training during the research protocol of 6 weeks.

### Statistical analysis

Data were presented as mean ± standard deviation (SD). Before the beginning of the study, researchers performed a test-retest reliability assessment (ICC, two-way mixed model) of each test between familiarization session and testing session (1 week distance) and interpreted as follows: > 0.9 = *excellent*; > 0.8 = *good*; > 0.7 = *acceptable*; > 0.6 = *questionable*; > 0.5 = *poor*; < 0.5 = *unacceptable* [30]. Intention to treat analysis was adopted (every participant was considered for the final analysis) [26, 27]. Shapiro-Wilk test was used for checking the normality (assumption) and Levene’s test for equality of variance (heteroscedasticity). Two-way analysis of variance (ANOVA) was employed to detect possible within-in and time*groups interactions [31]. Between-group differences were analyzed using the analysis of covariance (ANCOVA) using baseline values as covariate. Robust estimates of 95% confidence intervals (CI) were calculated using bootstrapping technique (randomly 1000 bootstrap samples). Mean difference estimate is based on the median of the bootstrapping distribution. Statistical significance was set at p < 0.05. Partial eta squared (η^2^) was calculated after ANOVA and ANCOVA and interpreted as follow, *trivial* η^2^ < 0.01, *small* η^2^ > 0.01, *moderate* η^2^ > 0.06, and *large* η^2^ > 0.14. Within effect was assessed using Cohen’s *d* and interpreted as *trivial* < 0.2, *small* = 0.2–0.6, *moderate* = 0.6–1.2, *large* = 1.2–2.0, *very large* > 2.0 [31]. Statistical analyses were performed by JASP software version 0.10.2 (Amsterdam, Netherland) for Macintosh.

## RESULTS

An attendance of 95% for both groups (TG and CG) was reported at the end of this study. Within-group variations after 6 weeks of training for both TG and CG are reported in Table 2.

**TABLE 2:** Summary of baseline and post-training data before and after 6 weeks of TG (n = 10) and CG (n = 10). Data are presented in mean ± SDs.

Variable	Baseline Mean ± SDs	Follow-up Mean ± SDs	Delta difference (95% CI)	P-level	Effect Size	Qualitative assessment
**TG**						
Sprint 10 m (s)	1.82 ± 0.06	1.74 ± 0.10	-0.08 (-0.18, 0.02)	0.100	-0.58	*Small*
Sprint 30 m (s)	4.28 ± 0.24	3.99 ± 0.10	-0.29 (-0.45, -0.12)	0.004	-1.12	*Large*
505-COD (s)	5.33 ± 0.18	5.19 ± 0.10	-0.13 (-0.25, -0.02)	0.024	-0.85	*Moderate*
Long Jump (cm)	222 ± 17	227 ± 19	4.6 (0.8, 8.4)	0.022	0.87	*Moderate*

**CG**						
Sprint 10 m (s)	1.92 ± 0.13	1.94 ± 0.16	0.02 (-0.09, 0.15)	0.658	0.14	*Trivial*
Sprint 30 m (s)	4.24 ± 0.29	4.42 ± 0.24	0.17 (-0.04, 0.40)	0.109	0.52	*Small*
505-COD (s)	5.52 ± 0.19	5.28 ± 0.20	0.03 (-0.05, 0.11)	0.453	0.25	*Small*
Long Jump (cm)	214 ± 15	212 ± 16	-1.9 (-5.2, 1.8)	0.299	0.35	*Small*

TG = Training group; CG = Control group; 505-COD = 505 change of direction test; SD = Standard deviations; CI = Confidence intervals; m = meters; s = seconds.

After 6 weeks of training, between-group analysis following ANCOVA using baseline values as covariate reported statistical difference in favor of the TG in sprint 10 m (F = 7.258, p = 0.015, η^2^ = 0.295, *large*), sprint 30 m (F = 27.719, p < 0.001, η^2^ = 0.599, *large*), in 505-COD (F = 5.942, p = 0.026, η^2^ = 0.154, *large*), and long jump (F = 6.004, p = 0.025, η^2^ = 0.027, *small*). Sprint 10 m reported a mean difference of -0.18 s, 95% CI (-0.36, -0.03), sprint 30 m reported a mean difference of -0.42 s, 95% CI (-0.61, -0.29), COD-505 reported a mean difference of -0.12 s, 95% CI (-0.25, -0.03), and LJ reported a mean difference of 5.9 cm, 95% CI (1.09, 10.4).

A time*group interaction was reported for 505-COD (F = 7.1, p = 0.016, η^2^ = 0.057, *small*) and long jump (F = 7.690, p = 0.013, η^2^ = 0.008, *trivial*).

## DISCUSSION

**FIG. 2:**
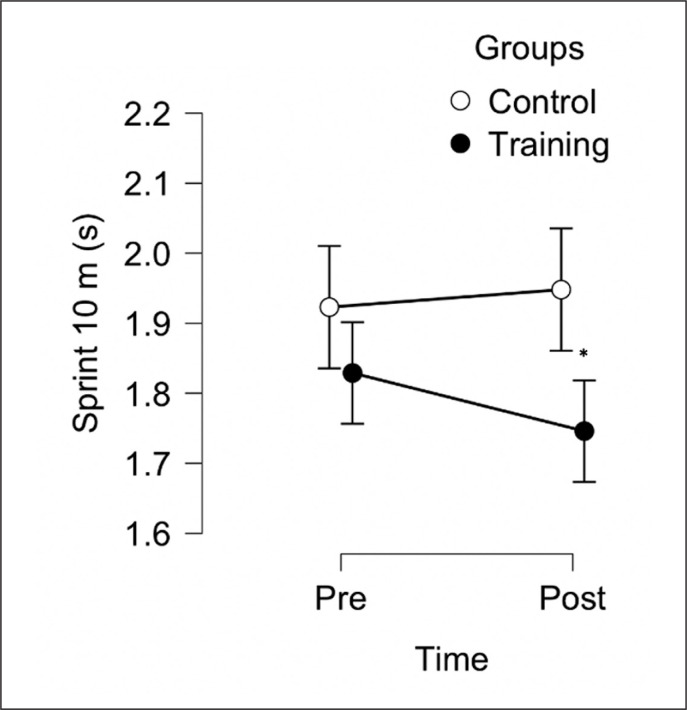
Between-group analysis after 6 weeks of Training Group (n = 10) and Control Group (n = 10). * Represents a significant difference (p < 0.05) between groups following analysis of convariance (ANCOVA) using baseline values as covariate.

The aim of this study was to evaluate the effect of 6 weeks, two sessions per week, of a TG (combined protocol, jump and sprint training) compared to CG on sprint 10 m, sprint 30 m, 505-COD and LJ performance in male soccer players. After the training period, TG reported some within group meaningful improvements for sprint 30 m, 505-COD and LJ, but not for sprint 10 m (see Table 1). Following between-group analysis, TG reported significant differences compared to the CG in all variables (Figure 2, Figure 4, Figure 4, Figure 5). These data indicate that combined sprint and jump training, when performed twice a week, in addition to the regular team training, can improve specific physical performance in male soccer players. Given that this training protocol leads to greater physical adaptations than regular soccer training, these findings support their combined use within soccer players training programs.

**FIG. 3:**
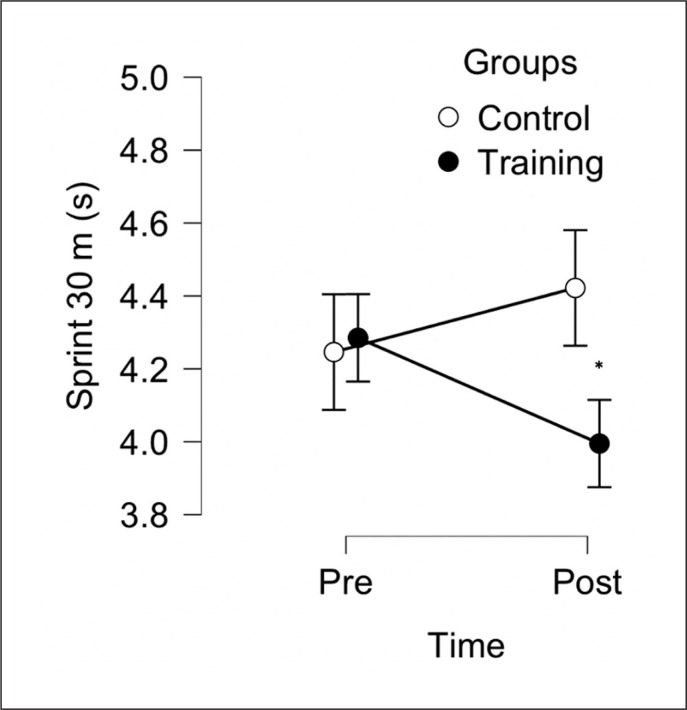
Between-group analysis after 6 weeks of Training Group (n = 10) and Control Group (n = 10). * Represents a significant difference (p < 0.05) between groups following analysis of convariance (ANCOVA) using baseline values as covariate.

**FIG. 4:**
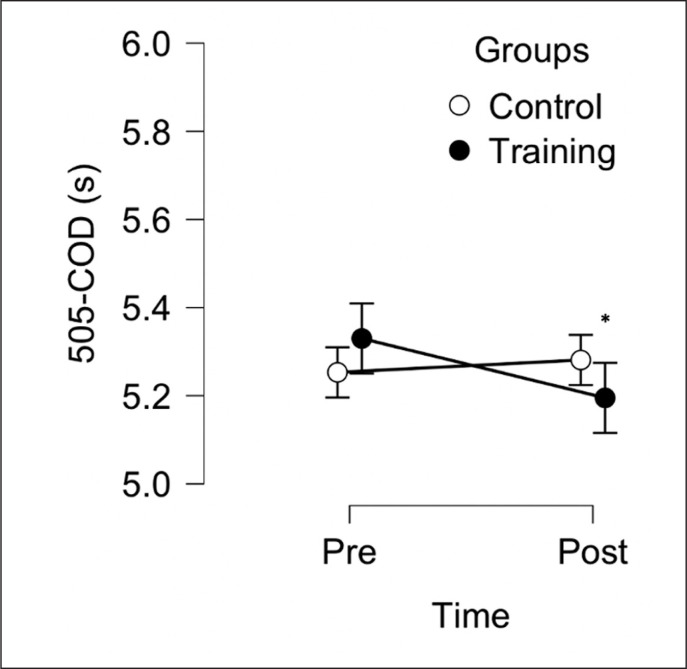
Between-group analysis after 6 weeks of Training Group (n = 10) and Control Group (n = 10). * Represents a significant difference (p < 0.05) between groups following analysis of convariance (ANCOVA) using baseline values as covariate.

**FIG. 5:**
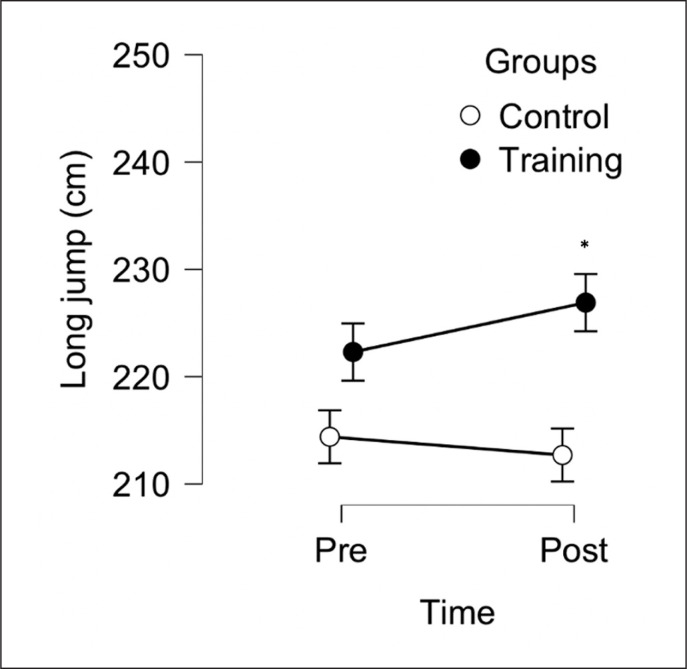
Between-group analysis after 6 weeks of Training Group (n = 10) and Control Group (n = 10). * Represents a significant difference (p < 0.05) between groups following analysis of convariance (ANCOVA) using baseline values as covariate.

The importance of improving sprinting, COD, and jump performance within professional soccer players to compete at high level is well known [3, 5, 6, 32]. However, it is not clear what is the most suitable approach to obtain the desired adaptations, in particular when training is performed in season with a limited time to dedicate to physical training [14, 33]. The protocol proposed in the current study used a training frequency of 2 sessions a week as it seems to be sufficient to induce physical adaptations in soccer players in-season in a relatively short period (i.e., 6–8 weeks) [14, 18, 22, 34], but not too demanding to interfere with the other training and coaching aspects such as technical and tactical training. In this study, we found meaningful between group differences in favor of the TG in sprint 10 m, sprint 30 m, COD and LJ performance, which considering the short-term protocol administered (6 weeks), may be primarily associated with neural adaptations (e.g., motor unit recruitment strategy and Hoffman reflex) [19, 35]. Neural adaptations are associated with improvement in maximal voluntary contraction, intermuscular coordination, stretch reflex excitability, and changes in leg muscle activation strategies [19]. Moreover, the combination of jumps included in this protocol, which have an emphasis on quick but high-intensity eccentric contractions (landing phase during the jump) and accelerations (which have a high neuromuscular load) may explain some of the adaptations found after only 6 weeks of training [36]. It is well known that the eccentric contraction can produce higher force levels (above isometric force capacities) than the concentric contraction [37], which could explain part of the improvements obtained in this study. Moreover, the advantages offered by the jump exercises are not only related to the eccentric demands, but the combination of eccentric and concentric phases, involving the SSC (muscle action when the muscles lengthening is immediately followed by muscles shortening), which allows elastic energy storage and consequently enhanced force and power production in the following concentric action [19, 38].

The development of short-distance (i.e., 5–20 m) sprint performance is a vital component of athletic performance within soccer [8, 9]. In the current study, there were some significant performance enhancements within the sprint 30 m (*large*) performance over the training period (Figure 3) as well as in in sprint 10 m (Figure 2) with a significant (p = 0015, *large*) between-group difference (however, we found only *small* but non-significant difference in the with-in analysis). Therefore, the results of this study are not surprising, given the recent meta-analysis by Nicholson et al. [8], who identified that combining training methods (e.g., sprinting and strength/ jump related exercises) is effective for developing short-sprint performance producing significant moderate-large standardized mean difference at 0–10 m (0.60 [95% CI 0.44, 0.75]) and 0–20 m (0.56 [95% CI 0.39, 0.74]). The explanation of these improvements could be associated with benefits offered by the combining sprint and jump training, as used in this study, which has been previously reported as capable of inducing improvements in acceleration performance [18, 34]. Reasons for this are unclear but it is possible that some neural adaptations from a combined training program are likely to improve short-sprint performance [8, 14, 21, 22].

In a systematic review [25], 24 studies suggested that plyometric training improves COD ability with a mean effect size range from 0.26 to 2.8. As aforementioned, following our combined protocol, we observed a significantly *moderate* improvements in 505-COD and LJ performance, which may be explained due to improved neural adaptations reported above (between group analysis reported in Figure 4 and Figure 5) [21] – although it is difficult to ascertain the exact physiological mechanisms since such data were not recorded in this study. However, in previous research that administered directional and jump training in youth soccer players, 505-COD did not improve [14]. Reasons for this are unclear however some methodological differences (in training design) could possibly explain such differences in findings between studies. Some previous studies used a lower training frequency and volume [14, 24] compared to the current study as well as training took place in a different moment of the season when players could have had different fitness levels. Finally, initial players’ training status and the contextual effect of team training with the administrated training protocols can play a key role in sport-specific physical adaptations [14, 24].

The positive results reported in this study are very important for practitioners because they show that a low volume of sprint and jump training, with a training frequency of twice a week (see Table 1), can be very beneficial for the improvement of sprint, jump and COD performance. Although this study found significant between-group differences in all the parameters analyzed, a higher volume protocol could have offered large benefits since adaptations (usually) follow a dose-response principle [19, 39]. Another important finding of this study is related to the positive sport specific improvements obtained during an in-season period since most intervention studies are performed in pre-season when more time is available for physical training. Throughout the annual soccer season, it is generally reported that there is a fitness improvement in preseason, with a maintenance of fitness in-season [40]. Subsequently, larger benefits of physical adaptation are expected for training programs administered in the preseason compared with in-season, when it is more difficult to elicit physical adaptations [19, 39]. Another strength of this study is the protocol used such as an RCT, which offers the highest scientific rigor.

### Limitations and future directions

Despite the novelty and practical application of the current study, this study is not without limitations. First, a larger sample size would allow further analysis of the effect of TG protocols on physical fitness, however this study is a reflection of the sample size available within this semi-professional soccer team. Second, this sample is only reflective of one male semi-professional team and, hence, may not be applicable to other specific populations such as professional male or female players. Third, this study is unable to ascertain if improvements in performances are mainly due to the sprint training, plyometric training, or the combination of the two training, however, this protocol was designed *a priori* to answer the needs of the club involved that was interested to understand if a combination of jump and sprint training was superior compared to soccer training alone – which was demonstrated. Future research should look to isolate specific training modalities within the same group of players to gain a greater understanding into the mechanisms of adaptation.

## CONCLUSIONS

This study indicates that combined sprint and jump training, when performed twice a week, for a duration of 6 weeks, in addition to the regular team training, can improve specific physical performance in male soccer players compared to a control condition (regular team training) and supports previous research findings. This study offers a unique practical application for programmes to be implemented in season such as combined sprint and jump activities are an effective training stimulus to improve sprint (e.g., 30 m), COD and jump performance in semi-professional soccer players. These training stimuli identify that it is possible to improve soccer players fitness levels in season in a short period of time (6 weeks). Fitness coaches and sports scientists can integrate their training proposals with the protocol described in this study (see Table 1). Specifically, a combination of 64–70 jumps and 675–738 m of sprinting training per session can yield such benefits. Finally, a volume increment of 10% after 3 weeks of training can be a suitable training dose progression.
